# Effects of 17-allylamino-17-demethoxygeldanamycin (17-AAG) in transgenic mouse models of frontotemporal lobar degeneration and Alzheimer’s disease

**DOI:** 10.1186/2047-9158-2-24

**Published:** 2013-12-17

**Authors:** Shuk Wai Ho, Yuk Tung Chanel Tsui, Ting Ting Wong, Stanley Kwok-Kuen Cheung, William B Goggins, Lau Ming Yi, Kwok Kin Cheng, Larry Baum

**Affiliations:** 1 Department of Surgery, The University of Hong Kong, Pokfulam, Hong Kong, China; 2 School of Biomedical Sciences (Pharmacology), The University of Nottingham, Nottingham, UK; 3 Department of Microbiology, The University of Hong Kong, Pokfulam, Hong Kong, China; 4 School of Public Health and Primary Care, The Chinese University of Hong Kong, Shatin, Hong Kong, China; 5 School of Pharmacy, The Chinese University of Hong Kong, Shatin, Hong Kong, China

**Keywords:** Dementia, Mouse, Tangles, Plaques

## Abstract

Alzheimer’s disease (AD), the most common dementia, is characterized by potentially neurotoxic aggregation of Aβ peptide and tau protein, and their deposition as amyloid plaques and neurofibrillary tangles (NFTs). Tau aggregation also occurs in other common neurodegenerative diseases. Frontotemporal dementia (FTD) can be caused by tau mutations that increase the susceptibility of tau to hyperphosphorylation and aggregation, which may cause neuronal dysfunction and deposition of NFTs. 17-allylamino-17-demethoxygeldanamycin (17-AAG) is a potent inhibitor of heat shock protein 90 (Hsp90), a cytosolic chaperone implicated in the proper folding and functions of a repertoire of client proteins. 17-AAG binds to Hsp90 and enhances degradation of Hsp90 client protein. We sought to determine whether 17-AAG can reduce Aβ and tau pathology in the brains of AD and FTD model mice expressing Aβ or P301L mutant tau, respectively. Mice were randomized to receive 25, 5, or 0 mg/kg 17-AAG thrice weekly from age eight to 11 months. Analysis was performed by rotarod test on motor function, on the area occupied by plaques in hippocampus or NFTs in medulla tissue sections, and on mortality. A high dose of 17-AAG tended to decrease NFTs in male mice (p = 0.08). Further studies are required to confirm the effect of 17-AAG in diseases of tau aggregation.

## Introduction

Abnormal aggregation of protein is a common theme among neurodegenerative diseases: Aβ peptide in Alzheimer’s Disease (AD), tau protein in AD and other tauopathies such as some forms of frontotemporal dementia (FTD), and α-synuclein in Parkinson’s disease and Lewy Body Disease [1-4]. These serious diseases occur with a high prevalence, yet no treatment is available to halt their progression, and such treatments are urgently needed. Drugs interfering with protein misfolding may hold promise for slowing neurodegenerative disease, as well as other disorders of protein aggregation [5-7].

Extracellular amyloid plaques and intracellular neurofibrillary tangles (NFTs) are the two core pathological lesions found in Alzheimer’s disease [3]. Amyloid plaques are mainly composed of Aβ, whereas NFTs consist of hyperphosphorylated tau. Aβ aggregation in the brain parenchyma may initiate a series of events that eventually results in AD dementia [2]. These events include activation of kinases that hyperphosphorylate tau, which may harm neurons by direct toxicity of abnormal tau or by decreased supply of tau to stabilize microtubules. Mutations in tau can also cause such dysfunctions, leading to frontotemporal lobar degeneration (FTLD) [1].

Tg2576 and JNPL3 are mouse models of Aβ and tau pathology, respectively [8,9]. Tg2576 mice contain the human APP gene with the Swedish mutation, leading to five-fold and 14-fold higher than usual levels of Aβ1-40 and Aβ1-42, respectively. Cognitive impairment develops at 9 to 10 months, with the presence of Aβ plaques in cortical and limbic regions. JNPL3 mice contain human tau P301L mutant protein, with expression controlled by the murine prion (PrP) promoter [10]. Similar hyperphosphorylated tau products are generated in both human tauopathies and JNPL3 mice [11]. This mutation causes motor and behavioural deficits, plus NFTs, starting at 4.5 months of age, in amygdala, medulla, cerebellum, hypothalamus, pons and spinal cord [8].

Therapeutic approaches include reducing the brain concentrations of Aβ and tau or inhibiting their aggregation [4,12,13]. Immunotherapy is being investigated as a method for removing Aβ or tau, either in any form or in aggregated forms. For example, JNPL3 mice immunized against tau oligomers showed slower disease progression and fewer tau aggregates than untreated mice [14]. It is possible that removal of aggregation-prone proteins might be even more effective if conducted before aggregation occurs, when the proteins are still monomeric. But removing normal forms of these proteins may disrupt their important cellular functions. Aggregation-prone forms of proteins may be identified by their abnormally folded structure.

Heat shock proteins (HSPs) are chaperone proteins that have the ability to remodel misfolded proteins or direct them to the degradation pathway under either normal or stress conditions. Since proteins must fold into precise three-dimensional conformations to achieve their biological functions, HSPs assist cells to defend against external stress, such as accumulation of misfolded proteins or inflammation [15-17].

Heat shock protein 90 (Hsp90) is one of the most abundant proteins in the cytosol of eukaryotic cells, accounting for 1-2% of the total proteins under non-stress conditions [18]. It has an essential role in the refolding of misfolded proteins under stress [19]. Despite this, inappropriate signal activation of Hsp90 could also occur during cellular stress and lead to toxic protein aggregation [20]. Hsp90 binds to a specific set of targets, which are known as Hsp90 client proteins. It interacts with its client proteins in a dynamic ATP dependent cycle to regulate folding and assembly of its clients, but the mechanism is poorly understood.

Hsp90 consists of three domains, a highly conserved N-terminal ATP-binding domain, a middle domain, and a carboxyl domain. Both the N-terminal and C-terminal domains play a role in preventing aggregation of denatured proteins *in vitro*. By inhibiting the N-terminal ATP binding domain, the activity of Hsp90 is reduced [21], suggesting that substrate binding is nucleotide regulated. Since Hsp90 has a unique N-terminal ATP binding site, high specificity inhibitors have been developed to inhibit the ATP-dependent chaperone activities of Hsp90 [22].

17-N-Allylamino-17-demethoxygeldanamycin (17-AAG), or tanespimycin, is a novel HSP90 inhibitor that has been extensively studied for targeting oncoproteins [23]. It is a less toxic derivative of geldanamycin (GA), a benzoquinoid ansamycin compound produced by *Streptomyces hygroscopicus* that has growth inhibitory activity against tumours [24]. However, due to undesired toxicity, GA was not suitable for clinical studies. Instead, 17-AAG has been or is now in clinical trials for a wide range of cancers. It is a candidate for treating neurodegenerative disorders.

17-AAG has two mechanisms of action. First, 17-AAG disrupts the function of Hsp90 by binding to its ATP binding pocket, inhibiting the formation of the stabilizing form and shifting the complex to the proteosome-targeting form, which leads to proteosome degradation of client protein [25]. Second, it inhibits the association of Hsp90 with heat shock factor-1, which results in transcription of chaperones such as Hsp70 and Hsp40 [20,26,27]. These two molecular chaperones act as protein quality control units, refolding misfolded proteins or leading them to destruction. They use the energy gained from ATP hydrolysis to bind and remodel substrates under conditions either with or without stress. Hsp70 requires assistance from Hsp40 to facilitate substrate recognition and stimulate the hydrolysis of ATP [28]. Induction of Hsp40 and Hsp70 by geldanamycin successfully suppressed mutant huntingtin in a Huntington’s disease cell model [20], suggesting the possibility of geldanamycin or 17-AAG as treatments for other neurodegenerative diseases involving protein misfolding. Waza et al. [15] tested 17-AAG to decrease mutant androgen receptor (AR), a client protein of Hsp90 that causes spinal and bulbar muscular atrophy (SBMA). Mutant AR products in cell models were drastically decreased after being treated by 17-AAG, even with complete inhibition of Hsp70 and Hsp40, which was achieved by protein synthesis inhibitor.

17-AAG might be used to block Aβ or tau aggregation. Hsp70 helps prevent early stages of protein aggregation, including that of Aβ [17]. It can function extracellularly [29] and therefore might promote disaggregation of extracellular Aβ. Hsp90 may play a role in Aβ peptide aggregation [17], and a non-toxic Hsp90 inhibitor significantly reduced Aβ neurotoxicity in embryonic primary neurons [30]. By targeting Hsp90, client proteins which are normally stabilized by Hsp90 will be targeted for degradation [31]. Therefore, 17-AAG could potentially act as a neuroprotective agent that counters the toxic effects of Aβ by disrupting its aggregation and promoting its clearance.

Inhibiting the action of Hsp90 may affect tau by several possible mechanisms. Tau is a Hsp90 client protein [32]. Hyperphosphorylated tau binds with higher affinity to Hsp90 than to normal tau [33]. Binding of hyperphosphorylated tau to Hsp90 enhances stabilization and aggregation [34,35], thus Hsp90 inhibition may reduce tau aggregation. Extracellular signal-regulated protein kinase (ERK) is an Hsp90 client protein, and 17-AAG is able to reduce the amount of and aggregation of phosphorylated tau, possibly by inhibiting the action of ERK [36]. 17-AAG is capable of inducing Hsp40 and Hsp70, of which Hsp70 was seen to be able to reduce the level of tau [33,37]. In light of the above evidence, 17-AAG is worth investigating as a therapeutic agent for tauopathies such as FTLD.

17-AAG has excellent bioavailability after intraperitoneal injection in mice [38], but oral bioavailability is poor. It undergoes extensive hepatic metabolism by cytochrome P450 (CYP) 3A4 and is widely distributed in body tissues [38,39]. Preclinical toxicology studies showed that 17-AAG has dose-limiting hepatic and gallbladder toxicity, but it is less toxic than geldanamycin [40]. Studies on the tissue distribution of 17-AAG in mice showed that it penetrates the blood brain barrier [41,42].

In this study, we investigated the effect of 17-AAG on Aβ aggregation in the Tg2576 transgenic mouse model of AD and tau aggregation in the JNPL3 mouse model of FTLD. We quantified the effects on several outcomes: mortality, motor function, and plaque and tangle pathology.

## Materials and methods

### Animals

Mice that overexpress Aβ or a mutant tau protein were bought from Taconic Farms, Inc. and were bred at the Chinese University of Hong Kong for experiments. The Chinese University of Hong Kong Animal Experimentation Ethics Committee approved the study. Tg2576 is a transgenic mouse model that is hemizygous for the human β-amyloid precursor protein gene with the Swedish mutation (K670N/M671L) (APPswe), which leads to Aβ overexpression. It was bred on F1 progeny of SJL/JcrNTac and C57BL6/NTac strains. Tau transgenic mice (JNPL3) were used as a tauopathy model. The mice are homozygous for the 4R0N isoform of the human microtubule-associated protein tau with the P301L mutation.

### Rotarod test

Motor function deteriorates in JNPL3 mice as they age, and this was assessed by rotarod test [43]. Four rounds of trial training and one round of testing were performed with a one hour interval in between. Mice were placed onto the rod of the apparatus. Rotation was started at 4 revolutions per minute (rpm) to allow the mice to balance and then was increased by about 1 rpm/9 seconds, to a maximum of 40 rpm. The time between the beginning of the acceleration of the rod and the falling of each mouse was recorded.

The time that each mouse could stay on the rotarod was measured three times before treatment and three times at the end of treatment. The three before-treatment times were averaged, as were the three after-treatment times. For mice that died during treatment, after-treatment time was assigned as missing rather than 0. These averaged times were then used as the outcome variable in a longitudinal data analysis using linear mixed models (multilevel models). The predictor variables were dose (0, 5, 25), condition (before treatment, after treatment), and the interaction between dose and condition. The interaction terms estimate the effect of the treatment, as they represent the difference between the dosage groups in change in times before and after treatment. Sex was also originally entered into the models but was found not to be a significant predictor of effect modifier. We analyzed the data again using a longitudinal analysis method (linear mixed model) which allows the use of all of the available times even when there is only one time for a mouse. This method models each time as a function of condition (before or after treatment), dose (0, 5, 25), and a condition x dose interaction. The interaction is essentially what tests the effectiveness of dose since it represents the difference between before and after times among the different dosage groups.

### Treatment with 17-AAG

Doses of 17-AAG were chosen based on a study to treat spinal and bulbar muscular atrophy in mice [15]. In that study, 2.5 mg/kg and 25 mg/kg 17-AAG were used, with 25 mg/kg producing better protection. Since the optimum dose was therefore likely closer to 25 mg/kg than to 2.5 mg/kg, we increased the lower dose for our study, choosing a level of 5 mg/kg.

17-AAG (Fermentek, Israel) was prepared as a 50 mg/ml stock solution in DMSO. Starting at age eight months, 48 Tg2576 and 94 JNPL3 mice were treated with 25, 5, or 0 mg/kg of 17-AAG in 100% DMSO (2 ml/kg) by thrice weekly intraperitoneal injections for 3 months before being sacrificed.

### Brain sample preparation

Mice were killed, and brains were removed and split into hemispheres. One hemisphere was immediately immersed in 13 mL ice-cold 10% neutral buffered formalin (10% NBF) and incubated at 4°C overnight. Fixed brains were processed according to standard immunohistochemical procedure. Paraffin sections of 8 μm were prepared from hippocampus, -1.7 mm to -.3 mm from the bregma, and from medulla, -5.8 to -6.0 from the bregma. Sections were then stained by Thioflavin S to detect amyloid plaques or by AT8 monoclonal antibody (1:50; Pierce Biotechnology) to detect neurofibrillary tangles.

### Thioflavin S staining

Thioflavin S staining was adapted from Westermark et al. [44]. 0.5 gram Thioflavin S was dissolved in 50 mL deionized water. The slides were immersed in Mayer’s hematoxylin for 5 min and then washed under gently running water for 5 min. The slides were placed into the Thioflavin S solution for 5 min and subsequently differentiated in 70% ethanol for 5 min. The slides were rinsed in distilled water, mounted in glycerin jelly, and stored in a dark chamber.

### Quantification of Aβ plaques

For the quantification of plaques [13,45], two Thioflavin S-stained serial sections per animal were used. For each brain section, four fields which contained the highest density of plaques were imaged and captured with a × 10, numerical aperture 0.5 objective using an Axioskop 2 plus ZEISS microscope (Carl Zeiss, Germany) with a fluorescein isothiocyanate filter (excitation at 488 nm). The total pixel area of plaques for each section was counted using SigmaScan Pro 5.0 software. The percentage plaque burden was calculated by dividing the total area of plaques by the total area of fields and multiplying by 100.

### AT8 immunohistochemistry

NFTs were detected by immunohistochemistry using AT8, a monoclonal antibody to a phosphorylated epitope on tau [46,47]. Slides with brain sections were de-paraffinized and rehydrated. Antigen retrieval was performed using citrate buffer. Slides were rinsed by PBS, and peroxidase block was applied. Slides were then blocked in 10% normal goat serum, and AT8 (1:50) antibody was subsequently applied and incubated overnight at room temperature.

Slides were rinsed with PBS, followed by incubation with goat anti-mouse IgG for 45 minutes (Dako Envision + HRP, Dako), and staining was developed using 3,3′-diaminobenzidine (DAB) peroxidase solution (Invitrogen). Slides were rinsed with PBS and counterstained with Harris Hematoxylin, dehydrated, and mounted.

### Quantitation of tangles

Methods to quantitate tangles on stained sections were adapted and modified from van Belle et al. and Zehr et al. [45,48]. Quantitation of tangles in immunostained sections was performed using the Axiovision 4 program. Only the medulla region was examined. For each mouse, two sections were randomly selected across the whole medulla and magnified by 40x under a microscope, and, for each section, four photos were taken of the fields with the highest density of NFTs. Thus, there were eight photos per mouse. SigmaScan Pro 5.0 software was used to detect pixels of immunoreactivity in each photo. The colour threshold was set manually to isolate the immunopositive area. The percentage of tau immunopositivity was calculated by dividing immunopositive pixels of each photo by the total number of pixels of the photo, giving one value per photo. The eight values of each mouse were averaged, giving one value per mouse. The software used was Microsoft Office Excel 2007.

### Statistical analysis

Results were analyzed by SPSS Statistics (Version 20). P < 0.05 was considered statistically significant. Data were presented in box plots with medians and interquartile ranges. The effect of 17-AAG on mortality was analyzed by Fisher’s Exact Test. Since the sample size was small, the data were not normally distributed, thus non-parametric analyses were used. The Mann–Whitney U test was used to analyze differences between treatment groups in total plaque area and area occupied by NFTs. Linear mixed models for longitudinal data were used to analyze the average rotarod times before and after treatment.

## Results

### Motor function

The model revealed that the rotarod performance of untreated mice (dose = 0) dropped an average of 39.7 seconds between before and after measurements (p = 0.0086). Times of low dose mice rose an average of 1.6 seconds between before and after measurements, and this was 41.3 seconds better than untreated mice (p = 0.052; this is the interaction p-value). Time of high dose mice dropped an average of 15.6 seconds between before and after measurements, and this was 24.2 seconds better than untreated mice (p = 0.22; this is the interaction p-value). The overall dose x condition interaction p-value = 0.14; this is the statistically most appropriate way to test the effectiveness of the treatment given no a priori ideas about which dose will work better. Thus, 17-AAG exhibited a tendency to protect motor function in JNPL3 mice.

### Mortality

**Figure 1:**
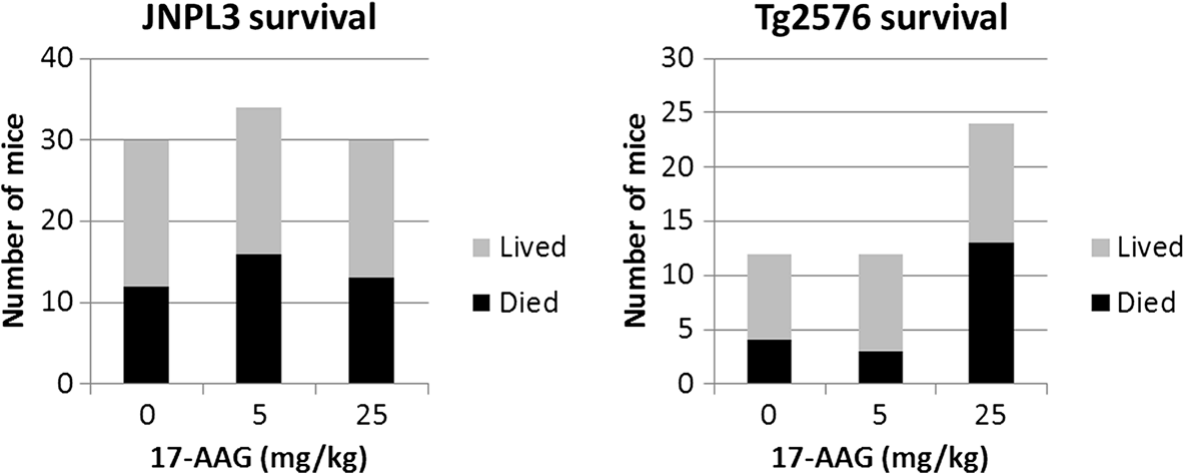
**Effect of 17-AAG on survival in JNPL3 and Tg2576 mice.** For each dose of 17-AAG, number of mice surviving to the end of the three-month treatment period at age 11 months or dying during treatment. Left: JNPL3 mice (tau transgenic). Right: Tg2576 mice (APP transgenic).

Treatment began on 48 Tg2576 and 94 JNPL3 mice at age eight months. Before treatment ended at age 11 months, 43% of mice died (Figure 1), however 17-AAG treatment did not significantly affect the death rate of either Tg2576 (p = 0.24, Fisher’s Exact Test) or JNPL3 (p = 0.84, Fisher’s Exact Test) mice. Autopsies were performed on some dead mice, revealing accumulations of fluid in the abdominal cavity and abnormal adhesions of intestines with membranes and muscles near the lower flank. In mice treated with 17-AAG, the fluid was purple or reddish-purple, which is the color of 17-AAG in DMSO. Mice completing three months of treatment received a cumulative intraperitoneal dose of DMSO of 7.8% (volume per body weight).

### Aβ deposition

**Figure 2:**
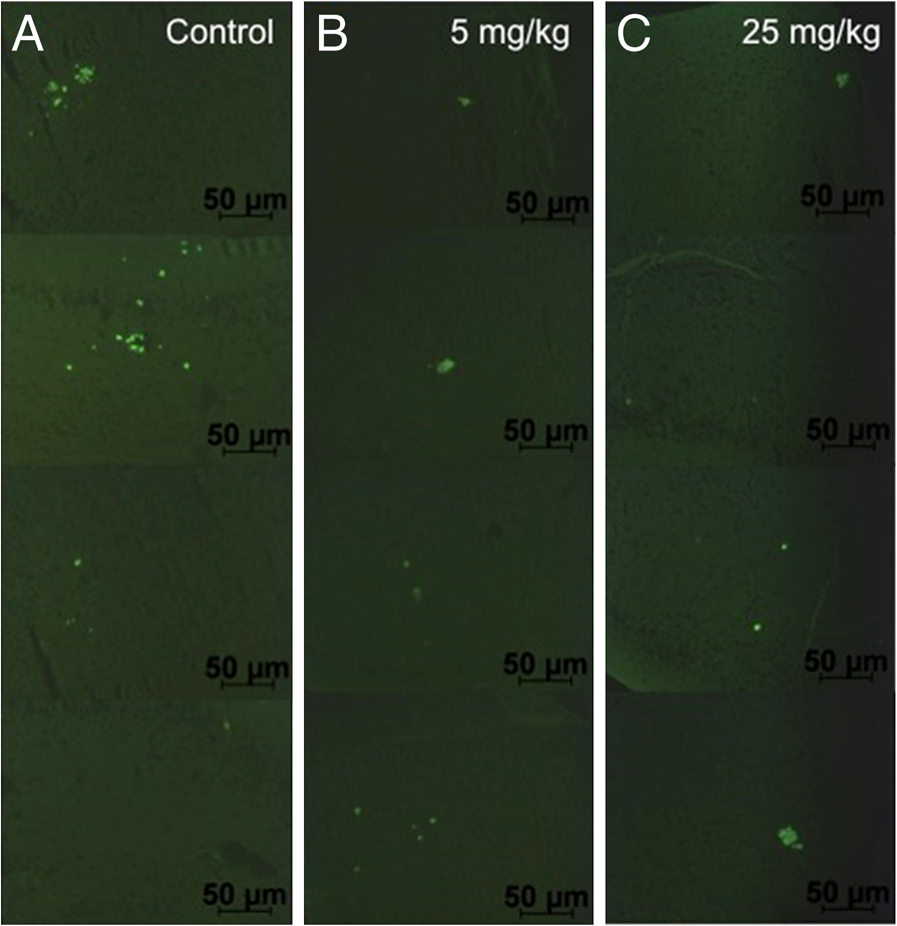
**Effect of 17-AAG on amyloid plaques in Tg2576 mice.** Thioflavin S staining of amyloid plaques (green) in the hippocampus of Tg2576 mice treated with different doses of 17-AAG: **A)** 0 mg/kg (Control), **B)** 5 mg/kg, **C)** 25 mg/kg. Four fields of each dose are shown.

Of the 24 Tg2576 mice completing treatment, brains of 5 were excluded due to errors or problems processing the tissue, and plaques were visualized in 19 mice by Thioflavin S staining of amyloid (Figure 2). Treatment with 17-AAG had no significant effect on the total plaque area (Mann–Whitney test comparing control with combined low and high dose: p = 0.97). Analyzing males and females separately also revealed no treatment effect on plaque area.

### Tau deposition

To quantify NFTs in JNPL3 mouse brains, immunohistochemical staining was performed using the AT8 antibody, which recognizes a phosphorylated epitope of tau. NFTs appeared brown. Haematoxylin was used as a counterstain of nucleic acid in cell nuclei.

**Figure 3:**
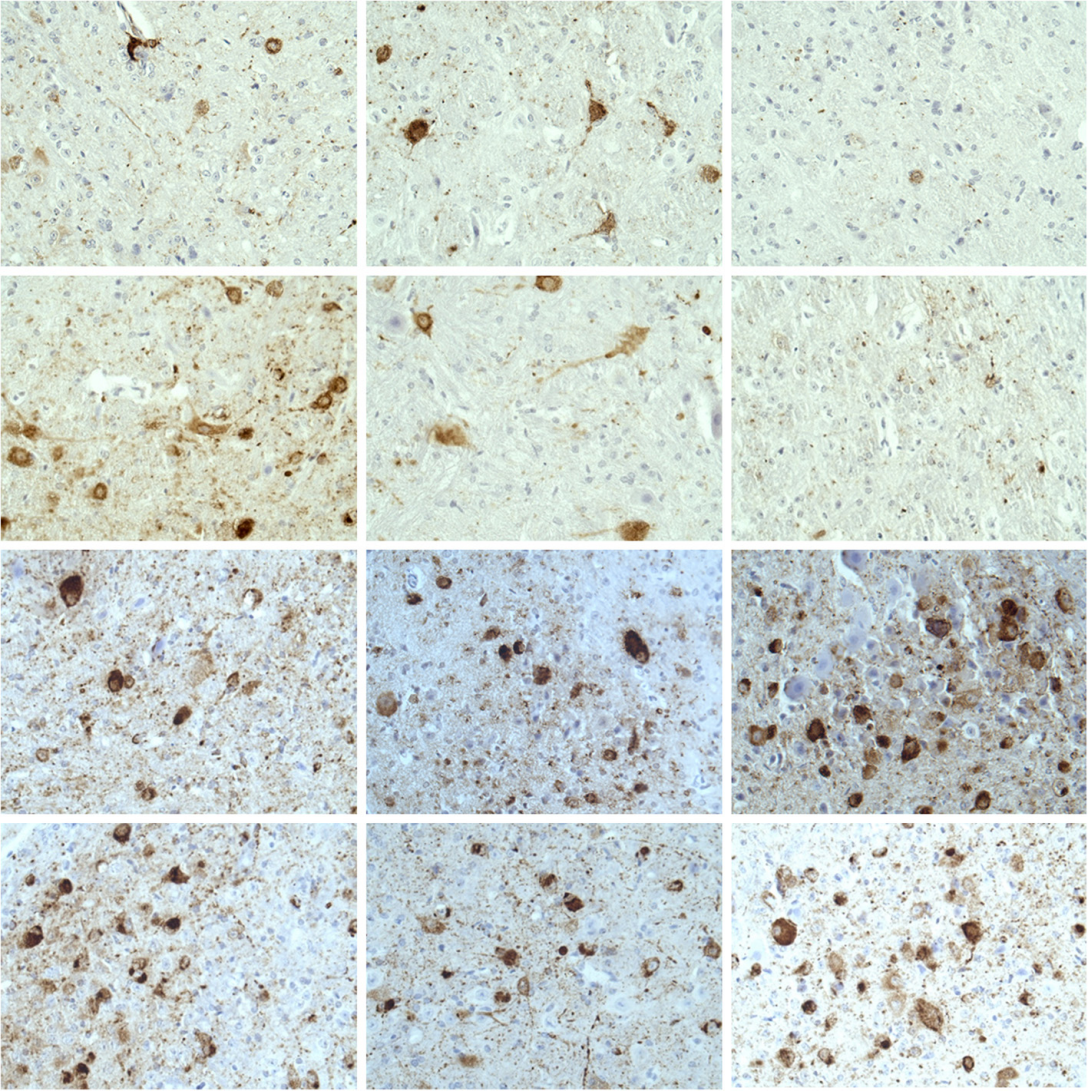
**Effect of 17-AAG on neurofibrillary tangles in JNPL3 mice.** AT8 immunostaining (brown) revealing NFTs in the medulla of JNPL3 mice treated with 17-AAG. Haematoxylin counterstain (blue) indicates cell nuclei. 40x magnification. Left: 0 mg/kg 17-AAG. Middle: 5 mg/kg 17-AAG. Right: 25 mg/kg 17-AAG. Top two rows: Male mice. Bottom two rows: Female mice.

**Figure 4:**
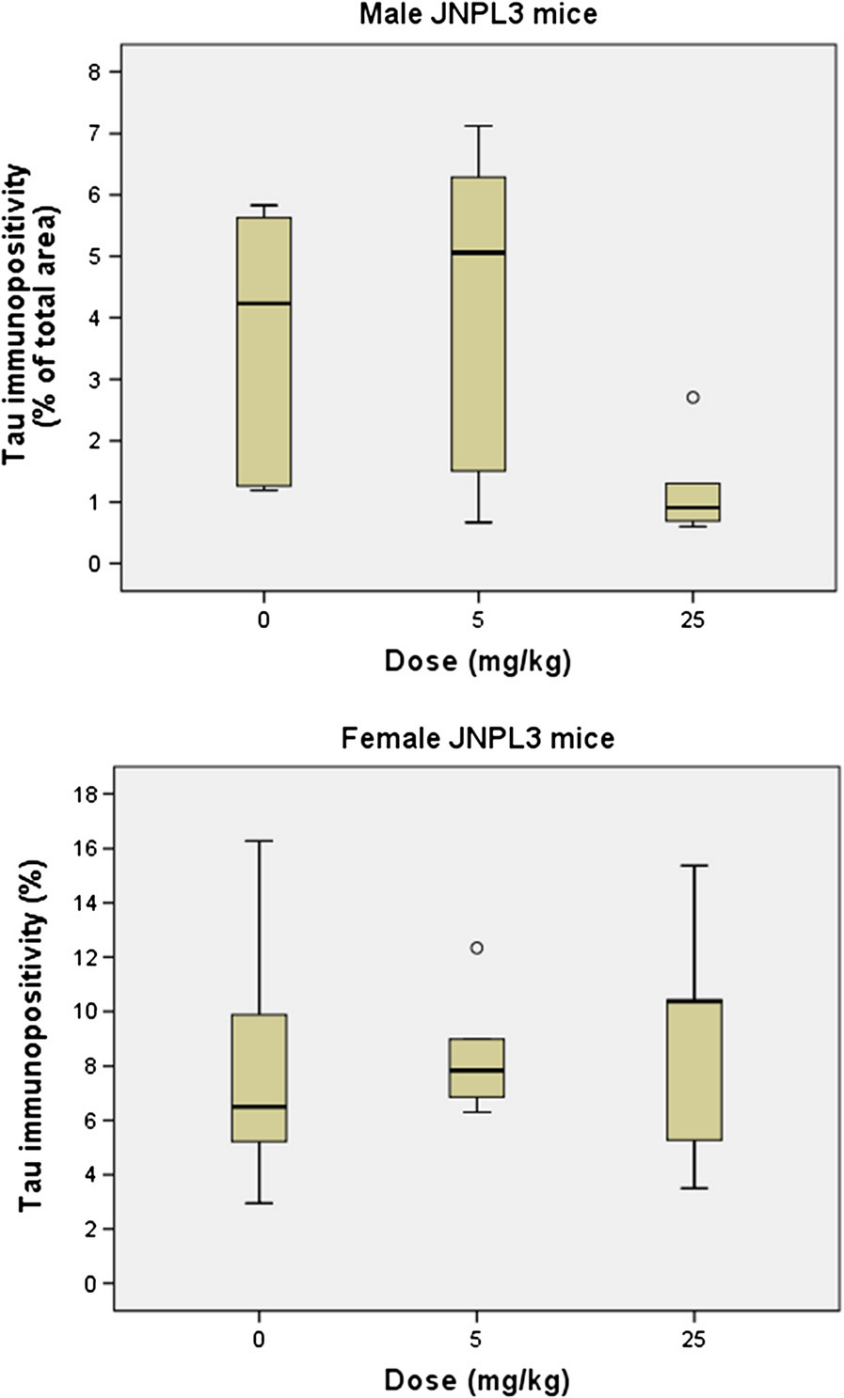
**Effect of 17-AAG on NFT area in JNPL3 mice.** Area of AT8 immunopositivity in the medulla of JNPL3 mice treated with 17-AAG. Boxes represent 25th and 75th percentiles; medians are the lines across the boxes; whisker caps represent 5th and 95th percentiles; and open circles are outliers. Doses of 17-AAG are shown as 0 mg/kg, 5 mg/kg, or 25 mg/kg. Top: Males. Bottom: Females.

The area occupied by NFTs did not differ between untreated mice (median area = 5.4%) and those receiving 17-AAG (either high or low doses; median area = 6.2%): p = 0.84 (Mann–Whitney U test). Figure 3 suggests that 17-AAG did not affect the number of NFTs in female mice, but that the highest dose may have reduced NFTs in males. Figure 4 displays area occupied by NFTs for each dose and sex. For males, Mann–Whitney U test resulted in p = 0.08 for high dose vs. no 17-AAG.

## Discussion

Many mice in the study died during treatment. However, mortality did not significantly differ between mice treated with or without 17-AAG, thus 17-AAG was not the cause of the high death rate. Perhaps repeated intraperitoneal injections, totaling 39 during the three month study, killed mice due to needle injury. However, other studies involving many intraperitoneal injections did not report adverse health effects on mice.

Alternatively, DMSO, which was used as the vehicle to dissolve 17-AAG, might have caused the deaths. As an effective solvent for a wide range of hydrophilic and hydrophobic compounds, DMSO readily penetrates animal tissue and is widely used as a solvent in research [49]. At injection sites, undiluted DMSO can irritate, inflame, and cause intravascular thrombosis [50]. For mice that completed the three month study, the cumulative dose of DMSO was 7.8% (v/w), quite a large proportion of body weight. Autopsies showed that the abdominal cavities of some of the mice contained liquid, purple or reddish-purple for mice receiving 17-AAG and clear for control mice, and revealed abnormal adhesions between membranes and muscles, which might have been caused by the solubilizing or inflammatory effect of exposure to accumulations of DMSO. An additional control group, using injections without either 17-AAG or DMSO, could have helped explore whether DMSO contributed to the early mortality we observed, however we did not anticipate this problem and thus did not plan a control group without DMSO.

DMSO was used because 17-AAG has limited water solubility. A potential alternative is Cremophor EL, but it may induce hypersensitivity reactions and anaphylaxis. Thus, patients require treatment with steroids and antihistamines before the administration of 17-AAG with Cremophor EL. When 17-AAG was used in a phase II clinical trial as an anti-tumour agent, only a limited effect of 17-AAG against tumors was observed, perhaps due to DMSO toxicity [51]. Thus, another clinical trial was conducted using Cremophor instead of DMSO, resulting in minimal toxicity [52]. Therefore, Cremophor, or safer delivery methods, may be considered for future studies.

Treatment with 17-AAG tended to preserve motor function in JNPL3 mice (p = 0.14), as measured by the rotarod test. Whether this tendency represents a real effect would require further studies, perhaps with a safer method of administration, larger numbers of mice, longer treatment, and treatment at different age ranges. It is possible that treatment began too late or did not last long enough to display effects to their greatest potential.

In this experiment, treatment with 17-AAG did not reduce Aβ deposition in a transgenic mouse model of Alzheimer’s disease. There are several possible explanations for this lack of an effect.

We had hypothesized that 17-AAG could up-regulate the expression of Hsp40 and Hsp70, which work synergistically to disaggregate and degrade misfolded proteins, and hence may suppress aggregation of Aβ. But 17-AAG had limited ability to induce Hsp40 and Hsp70 *in vivo*[20,53]. Induction of these heat shock proteins may have been insufficient.

Another possibility is that Aβ may not be a Hsp90 client protein. Hsp90 assists protein folding and stabilization of its client protein, and 17-AAG is an Hsp90 inhibitor. Although Hsp90 co-localized *in vivo* with amyloid filaments, indicating that Hsp90 may have a role in either promoting or suppressing the refolding of misfolded amyloid proteins [17,54], the overall effect of Hsp90 on Aβ aggregation is not clear. Since the action of Hsp90 is ATP-dependent, and since the increase of reactive oxygen species induced by Aβ may interfere with cellular mitochondrial energy metabolism, thereby decreasing the supply of ATP [55], Aβ might partially inhibit its own aggregation by inhibiting Hsp90. When taking into account the possible contradictory effects on protein aggregation, it is hard to predict whether 17-AAG would raise or lower the level of protein aggregates.

Another possible reason may be that the mice were too old when they began treatment. Inhibition of intracellular Hsp90 may prevent misfolding of Aβ monomers, but might not be effective to reverse extracellular aggregation of Aβ once it has occurred. An earlier treatment may attenuate the Aβ aggregation before pathology begins, but mice of younger ages would be needed to test this.

Alternatively, the number of mice may have been insufficient. Detection of a small treatment effect would require many mice.

Finally, the method of quantification might have obscured a treatment effect. By selecting the microscope fields with the highest concentrations of plaques, a treatment effect that decreased maximum or overall plaque density would be detected, but an effect only on areas of lower plaque density would be missed, as would an effect on Aβ oligomers but not on plaques.

Tau is a member of the Hsp90 client protein family, and mutant or hyperphosphorylated tau binds to Hsp90 at high affinity compared with normal tau [32]; therefore, theoretically, Hsp90 inhibitor might promote degradation of hyperphosphorylated tau and hence reduce neurotoxicity and decrease NFT formation. However, among all mice in our study, 17-AAG did not significantly reduce tau deposition.

As for NFT quantification, the method used to quantify NFTs might have obscured a treatment effect. By selecting the microscope fields with the highest concentrations of NFTs, a treatment effect that decreased overall or maximum NFT density would be detected, but an effect only on areas of lower NFT density would be missed, as would an effect on tau oligomers but not on NFTs. Quantifying total tau rather than only hyperphosphorylated tau may also have provided additional data, however hyperphosphorylated tau is a marker of dysfunction and aggregation in tauopathies and is thus more informative.

It is notable that, although 17-AAG did not decrease NFTs when all mice were examined together, when mice were analyzed by sex, male but not female mice exhibited a tendency toward a decrease in NFTs with the high dose of 17-AAG. A possible explanation could be that female JNPL3 mice tend to develop phenotypic features earlier than male JNPL3 mice, as found in previous research [56] and in the current study, in which 11-month old female mice displayed more NFT than did males of the same age. Sex differences have also been found in other amyloidoses [57-59]. If treatment with 17-AAG is effective at early stages of tauopathy, it would be more beneficial in male than female mice of the same age. A possible explanation for the difference in tauopathy progression between male and female mice is the difference in hormonal states. The primary female steroid sex hormones are estrogens, which occur as three types: estrone, estradiol, and estriol, of which estradiol is the most potent. Numerous studies, including both human and animal, have shown that estrogens can be neuroprotective and neurotrophic [60-62], though studies also suggest estrogens may either reduce or increase AD risk [63]. Estradiol can decrease tau phosphorylation [64]. Yet female JNPL3 mice display more NFTs than males of the same age. The mechanism for this difference remains unknown.

Luo et al. [34] tested the effect of an Hsp90 inhibitor on tau reduction, both *in vitro* and *in vivo*. As in the present study, they used Hsp90 inhibitors and JNPL3 mice. Their mice were heterozygous females aged 6.5 months, treated five times per week for 30 days. The Hsp90 inhibitors, PU24FC1 and PU-DZ8, reduced tau expression and phosphorylation (p = 0.0034). The team concluded that Hsp90 inhibitors can prevent and reverse tau aggregation. However, we found no effect of Hsp90 inhibition on tau pathology in female JNPL3 mice. Differences in the mice between the two studies might explain the discrepancy. The Luo mice had milder pathology because they were younger (7.5 months vs. 11 months at the end) and expressed less mutant tau (hemizygous vs. homozygous). Consistent with our finding of a tendency toward a protective effect of 17-AAG in the subset of mice with milder pathology—males—the drug might be useful against tauopathies at early stages of disease. Further studies may explore this possibility.

## Competing interests

The authors declare no competing interests.

## Authors’ contributions

SWH performed part of the mouse experiments and processed the brain tissue. YTCT performed AT8 immunohistochemistry and analysis and drafted part of the manuscript. TTW performed Thioflavin S staining and analysis and drafted part of the manuscript. SKKC performed part of the mouse experiments. WBG performed statistical analysis of rotarod data. LMY helped with mouse experiments. KKC supervised AT8 and Thioflavin S staining and analysis. LB designed and supervised the study and drafted part of the manuscript. All authors read and approved the final manuscript.
